# Therapeutic Approaches Targeting PAX3-FOXO1 and Its Regulatory and Transcriptional Pathways in Rhabdomyosarcoma

**DOI:** 10.3390/molecules23112798

**Published:** 2018-10-28

**Authors:** Thanh Hung Nguyen, Frederic G. Barr

**Affiliations:** Laboratory of Pathology, National Cancer Institute, 10 Center Drive, Bethesda, MD 20892, USA; thanh.nguyen5@nih.gov

**Keywords:** rhabdomyosarcoma, oncogenic transformation, gene fusion, transcription factor, targeted therapy

## Abstract

Rhabdomyosarcoma (RMS) is a family of soft tissue cancers that are related to the skeletal muscle lineage and predominantly occur in children and young adults. A specific chromosomal translocation t(2;13)(q35;q14) that gives rise to the chimeric oncogenic transcription factor PAX3-FOXO1 has been identified as a hallmark of the aggressive alveolar subtype of RMS. PAX3-FOXO1 cooperates with additional molecular changes to promote oncogenic transformation and tumorigenesis in various human and murine models. Its expression is generally restricted to RMS tumor cells, thus providing a very specific target for therapeutic approaches for these RMS tumors. In this article, we review the recent understanding of PAX3-FOXO1 as a transcription factor in the pathogenesis of this cancer and discuss recent developments to target this oncoprotein for treatment of RMS.

## 1. Introduction

Rhabdomyosarcoma (RMS) is a heterogeneous group of malignant soft tissue tumors that share biological features with skeletal myogenesis. Although considered a rare malignancy, RMS is one of the most common cancers in children, accounting for approximately 50% of all soft tissue sarcomas or ~3–8% of all pediatric cancers [1,2,3,4,5]. 

RMS tumors are traditionally divided into two major subtypes, embryonal RMS (ERMS) and alveolar RMS (ARMS), based on their histologic features [6,7,8,9]. ERMS is the most common subtype, accounting for approximately 70–80% of RMS cases, while ARMS represents about 20–30% of RMS cases. Each histologic subtype is associated with distinct genetic alterations (Table 1). Point mutations, often involving genes encoding proteins in the RAS signaling pathway, are frequently found in ERMS tumors whereas PAX3-FOXO1 (~60%) and PAX7-FOXO1 (~20%) gene fusions are hallmarks of ARMS tumors [10,11,12,13,14,15] and reviewed in [9,16]. Other fusions of PAX3 with nuclear proteins such as FOXO4 and NCOA1 have been found in rare cases of ARMS tumors [17,18,19,20,21,22,23]. The remaining 20% of ARMS cases do not have any detectable gene fusions, and show mutations characteristic of ERMS tumors [18,24]. In contrast, these recurrent point mutations are rare in fusion-positive ARMS cases. In recent literature, RMS tumors are often divided into PAX gene fusion-positive (FP) and fusion-negative (FN) categories [9,17]. This fusion-based classification seems to reflect better the tumor genetics, which may eventually guide targeted therapeutic decisions, and clinical course of RMS tumors. Several clinical studies provide evidence that this genetic stratification more accurately predicts the clinical progression, treatment response and prognosis of RMS patients [12,18,24,25,26,27,28]. ERMS tumors and FN tumors with an ARMS appearance are generally associated with a good outcome whereas FP tumors are more aggressive, often metastatic, less responsive to chemotherapies, frequently recurrent and have a worse prognosis [12,24,26,29,30,31]. Furthermore, in the setting of FP tumors, patients with PAX3-FOXO1-positive tumors have a worse outcome than those harboring the PAX7-FOXO1 fusion [27,28]. 

Current management of RMS is based on multimodal treatment that includes surgery and traditional chemotherapy with or without radiotherapy [32,33,34,35]. During the last few decades, effective regimens were identified and have contributed to significantly improved survival. The current chemotherapy regimen for RMS patients relies on a three-drug backbone of vincristine, actinomycin D and cyclophosphamyide [36]. Over the last 30–40 years, the 5-year survival rate of RMS patients has increased to over 60% [2,37]. However, the improved outcome mostly benefits ERMS patients, who have a 5-year survival over 70%, in contrast to ARMS patients, who have a 5-year survival of less than 50% [2]. In addition, though these cytotoxic drugs effectively control most localized RMS cases, the treatment is not very effective against metastatic cases. Approximately 12% of all RMS and 24% of ARMS cases present with distant metastases at the time of diagnosis [38]. In the group of metastatic ARMS tumors, the 5-year overall survival falls below 20% [38]. Many ARMS patients may be initially responsive to multimodal therapy, but later recur and develop metastases. In addition to the ineffectiveness of current therapies in several RMS subsets, many RMS survivors suffer from immediate and long-term treatment-associated toxicities [39,40], underlining the need for more specific drugs. 

Molecularly targeted chemotherapy has proven to be successful for treatment of many types of cancer. The finding of RMS subsets harboring distinct genetic alterations provides the starting point for the development of targeted therapy that can be individualized for these RMS subsets. In this article, we focus our review on PAX3-FOXO1 and its signaling as molecular points for targeting FP RMS tumors. 

## 2. PAX-FOXO1 Fusions are Oncogenic Drivers and Therapeutic Targets in FP RMS Tumors 

**Figure 1:**
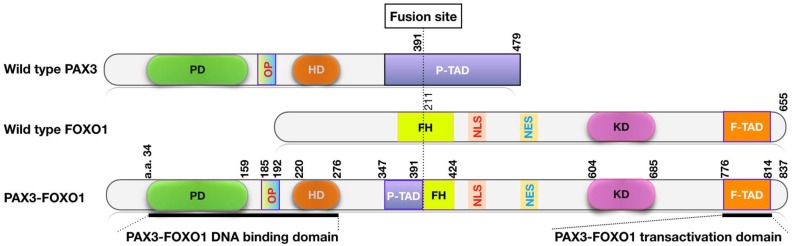
Protein domain structures of the wild-type PAX3, FOXO1 and PAX3-FOXO1 fusion proteins. The numbers indicate the corresponding amino acids present on each protein. Abbreviations: PD, Paired box domain; OP, octapeptide motif; HD, homeobox domain; P-TAD, PAX3 transactivation domain; FH, forkhead domain; KD, KIX-binding domain; F-TAD, FOXO1 transactivation domain; NLS, nuclear localization signal; NES, nuclear export signal.

The PAX3-FOXO1 and PAX7-FOXO1 fusion genes are products of the characteristic translocations t(2;13)(q35;q14) or t(1;13)(p36;q14), respectively. The fusion genes are transcribed into fusion transcripts that are in turn translated in fusion proteins. These events result in an in-frame fusion of the DNA binding domains of paired box family protein PAX3 or PAX7 to the transcriptional activating domain of the forkhead family protein FOXO1 (Figure 1) [16,41,47,48]. 

PAX3 and PAX7 are transcription factors that play essential roles during myogenesis [49]. The PAX3-FOXO1 and PAX7-FOXO1 fusions, which contain intact DNA binding domains from these PAX proteins, are much more potent than wild-type PAX3 or PAX7 in activating transcriptional target expression [50] (Figure 1). Transcriptomic analysis revealed that PAX3 and PAX3-FOXO1 regulate overlapping sets of target genes, although some genes may be preferentially regulated by the fusion [51]. Several lines of evidence indicate that the PAX gene fusions, in cooperation with other genetic changes, are oncogenic drivers in FP RMS tumors. First, the fusion transcription factors induce expression of a number of transcriptional targets that promote oncogenic transformation, including MET, ALK1, MYCN, IGFR1 and FGFR4. Second, FP RMS tumors have a significantly lower number of somatic mutations overall, and few if any recurrent mutations, as compared to FN RMS samples [17]. Third, PAX3-FOXO1 expression is capable of driving oncogenic transformation in cell culture and animal models. Targeted conditional expression of PAX3-FOXO1 in the myogenic lineage of mice induces the formation of tumors resembling human ARMS [52,53]. Furthermore, inactivation of CDNK2A and/or TP53 substantially augments the frequency and progression of these PAX3-FOXO1-driven tumors [52], indicating a possible cooperation of these genetic elements during tumorigenesis. In human cells, PAX3-FOXO1 stimulates cell growth and proliferation in a number of cell culture and xenograft tumor models [54,55,56,57,58,59]. Although expression of PAX3-FOXO1 alone is not able to induce transformation of normal human cells [60,61,62], it can cooperate with additional events, including inactivation of CDKN2A and overexpression of TERT and MYCN to promote tumorigenesis [52,55,62,63]. Similar changes are observed in human RMS tumors [64,65,66,67,68]. In addition to its tumorigenic activity, PAX3-FOXO1 also enhances cell invasion and migration [53,54,57,69]. Fewer experiments have been conducted to examine the role of the closely related PAX7-FOXO1 protein. However, given the similarity in protein structure and activity of the PAX fusions [70], it is expected that the two fusions exert similar effects in promoting tumorigenesis. In addition to the above-described experiments with enforced aberrant PAX3-FOXO1 expression, complementary experiments (described below) in which PAX3-FOXO1 is depleted have shown that PAX3-FOXO1 loss leads to growth arrest and stimulates terminal myogenic differentiation and apoptosis of FP RMS cell lines [54,55,58,59,71]. These results confirm the pro-cancer activity of PAX3-FOXO1 and provide a proof of principle that it is a good cancer target.

The formation of an invariant PAX3-FOXO1 fusion in the majority of ARMS tumors further enhances its value as an attractive target for cancer treatment. It is interesting to note that the PAX3-FOXO1 fusion has been found to be temporarily expressed at the mRNA and protein level during some early stages of normal myogenesis, perhaps as a result of trans-splicing of the PAX3 and FOXO1 transcripts [72,73]. However, it appears that PAX3-FOXO1 is only capable of interfering with normal myogenesis and promoting oncogenic transformation when constantly expressed as a result of a genetic rearrangement [72]. Furthermore, this aberrant fusion has not been detected in normal myogenic cells in children and adults, who would be the patients treated with such targeted therapy. Therefore, the specificity of targeting only tumor cells in these patients is another important aspect of this approach since normal cells will not be significantly affected by many of the proposed interventions.

## 3. Pharmaceutical Targeting of PAX3-FOXO1 Regulatory and Transcriptional Pathways 

### 3.1. Direct Inhibition of PAX3-FOXO1 by Small Molecules

Design and development of small molecule inhibitors have resulted in remarkable progress for treatment of certain cancers, particularly with drugs targeting protein kinases. However, little progress has been made in directly targeting many transcription factors, some of which appear to be potentially attractive cancer targets, such as PAX3-FOXO1. Direct inhibitors are expected to work by (i) specific binding and promotion of degradation and/or (ii) specific binding and blocking sites required for target protein activation or interaction with other critical effector proteins. The inability to date to design direct inhibitors for wild-type and fusion transcription factors can be attributed in part to the large protein-protein interaction interfaces and absence of deep protein pockets that are common targetable sites for drug design in the case of protein kinases [74,75]. Furthermore, the greater post-translational protein stability of PAX3-FOXO1 compared to the wild type PAX3 [76] can also be an additional obstacle for strategies promoting degradation of the PAX3-FOXO1 protein.

### 3.2. Inhibition of PAX3-FOXO1 Regulatory Networks

Given the challenges associated with modeling and designing direct inhibitors for PAX3-FOXO1, other strategies have been explored. There has been significant progress in several approaches, such as targeting signal transduction pathways that enhance stability and activity of PAX3-FOXO1, and inhibiting its transcriptional co-activators. Disruption of these pathways or coactivators leads to selective suppression of cell growth and proliferation of PAX3-FOXO1-expressing ARMS cell lines, and thus represents attractive therapeutic strategies. 

#### 3.2.1. Targeting Phosphorylation of PAX3-FOXO1

**Figure 2:**
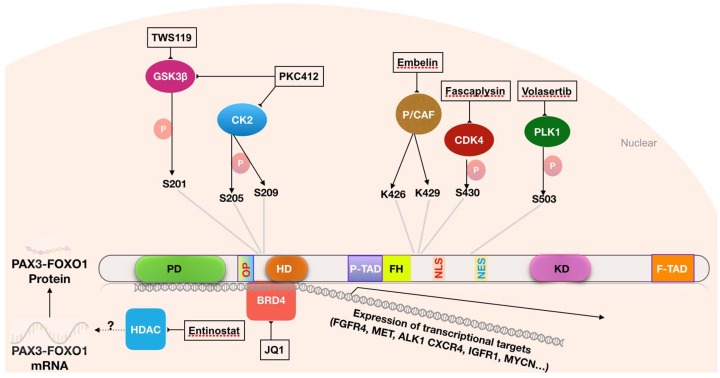
Targetable regulations of PAX3-FOXO1 activity. GSK3β, CK2, CDK4 and PLK1 phosphorylate PAX3-FOXO1 at various serine residues. The acetyltransferase enzyme P/CAF acetylates PAX3-FOXO1 at K426/K429 lysine residues. BRD4 acts as co-transcriptional activator of PAX3-FOXO1 at its DNA target sites. The mechanism by which HDAC regulates the mRNA expression level of PAX3-FOXO1 remains unknown. These chromatin modifiers, which modulate expression of PAX3-FOXO1 target genes, can be targeted by small molecule inhibitors.

Phosphorylation of PAX3-FOXO1 modulates its transcriptional activity and protein stability (Figure 2) [77,78,79,80]. This modification is carried out by protein kinases, which is a class of proteins that represents a large proportion of actionable cancer targets and for which inhibitors have been intensively screened and developed. The widespread generality of these phosphorylation pathways is particularly important to provide potential drugs to interrogate FP RMS and other rare cancers with critical targets regulated by phosphorylation. Thus, understanding the biology of PAX3-FOXO1 may provide the opportunity to utilize the existing pool of specific inhibitors or drugs to suppress PAX3-FOXO1 activity. Towards this goal, RNA interference and chemical screens have been conducted to identify kinases that promote PAX3-FOXO1 activity and their inhibitors (Table 2).

Several serine/threonine protein kinases phosphorylate PAX3-FOXO1 at serine residues and influence its transcriptional activity (Figure 2). Serine residue 201 (S201), which is located within the PAX3 DNA binding domain, is phosphorylated by GSK3β [81], while neighboring residues S205 and S209 are modified by casein kinase II (CK2) [82]. Serine 430, which is located within the FOXO1 region, is phosphorylated by CDK4 [79]. These modifications lead to enhanced transcriptional activity of the fusion protein, promoting the expression of its downstream targets and oncogenic effects. In addition, inhibition of GSK3β, CK2 and CDK4 suppresses transcriptional activity of PAX3-FOXO1, and in the case of serine 430, inhibition of phosphorylation has been reported to lead to cytoplasmic localization of the protein [78,79,81,82,83]. Similarly, a kinome-targeted siRNA screen found Polo-like kinase-1 and 4 (PLK1 and PLK4) as PAX3-FOXO1 upstream regulators. These Polo-like kinases phosphorylate PAX3-FOXO1 at serine 503 (S503), resulting in enhanced protein stability and oncogenic activity [80]. Concurrently, a parallel small molecule screen identified that PLK1 inhibitors, BI-2536 and BI-6727, strongly inhibited transcriptional activity of PAX3-FOXO1.

These findings raise the possibility that inhibition of PAX3-FOXO1-modifying kinases may be a useful strategy in the treatment of FP RMS. It should be noted that inhibitors of these kinases are already being actively studied in other cancers [89,90,91]. Several GSK3 inhibitors, including TWS119, SB216763, TDZD-8 and roscovitine, suppress PAX3-FOXO1 transcriptional activity, inhibit proliferation and induce apoptosis of PAX3-FOXO1-expressing cells [78]. Similarly, fascaplysin, a selective inhibitor of CDK4/Cyclin D1, also inhibits PAX3-FOXO1 transcriptional activity, and exerts a superior growth inhibitory effect in FP compared to FN RMS cells [79]. Another small molecule, PKC412, which targets a group of protein kinases, inhibits phosphorylation of PAX3-FOXO1 at multiple serine residues (S187, S193, S197, S201, S205 and S209), and suppresses growth in FP RMS lines more effectively than in FN RMS lines in both in vitro and in vivo assays [77]. The PLK1 inhibitors, BI-2536 and BI-6727, promote degradation of the PAX3-FOXO1 protein and induce regression of FP RMS xenogratft tumors [80]. In general, though these kinase studies suggest potential utility as a strategy for FP RMS treatment, the specificity and selectivity for PAX3-FOXO1-expressing cells need to be more fully investigated and independently reproduced in additional preclinical studies.

The sarco/endoplasmic reticulum Ca^2+^ ATPase inhibitor, thapsigargin, is another potentially useful drug that suppresses expression of PAX3-FOXO1 targets and induces apoptosis in FP RMS cell lines both in vitro and in vivo [84]. The inhibitory effect of thapsigargin on PAX3-FOXO1 transcriptional activity is accompanied by AKT activation, increased phosphorylation of PAX3-FOXO1 and decreased PAX3-FOXO1 protein expression. Although PAX3-FOXO1 contains two of the three serine residues phosphorylated by AKT1 in wild-type FOXO1 (S256 and 319), a previous study showed that AKT1 decreased the transcriptional activity of wild-type FOXO1 but did not affect the transcriptional activity of PAX3-FOXO1 [92]. Thus, it remains unclear how thapsigargin suppresses PAX3-FOXO1 activity. Moreover, the thapsigargin-induced activation of the usually pro-oncogenic AKT signaling pathway might be a concern for cancer therapy. 

#### 3.2.2. Targeting Transcriptional Co-Activators of PAX3-FOXO1

Recent studies suggest that targeting co-transcriptional activators of PAX3-FOXO1 is another potential approach for inhibiting PAX3-FOXO1 activity. The function of most transcription factors depends on direct interactions with cofactors as a means of influencing the transcription machinery to promote expression of target genes [93]. Several studies provide evidence that PAX3-FOXO1 needs binding partners to activate transcription of its downstream effectors. One such important co-regulator of PAX3-FOXO1 transcriptional activity is the chromodomain helicase DNA binding protein CHD4 [94]. PAX3-FOXO1 recruits CHD4 to activate expression of a subset of PAX3-FOXO1 target genes that promote cell proliferation. Inhibition of CHD4 reduces viability of FP but not FN RMS cells in vitro and in xenograft tumors [94]. In addition, PAX3-FOXO1 partners with several other proteins such as BRD4, MED1 and p300. Through interactions with these proteins, PAX3-FOXO1 co-occupies and establishes myogenic super enhancers on target genes, such as FGFR4, MYCN, ALK and MET, whose expression has been shown to be responsible for oncogenic activities of PAX3-FOXO1. BRD4 interaction is required for stability and functionality of PAX3-FOXO1 at these key super enhancers, and BRD4 inhibition suppresses PAX3-FOXO1-driven cell growth in FP RMS cell lines in vitro and in xenograft tumors [85]. 

It should further be noted that both CHD4 and BRD4 are epigenetic chromatin modifiers. CHD4 is an integral component of the nucleosome remodeling deacetylase (NuRD) complex that possesses both chromatin remodeling activity with histone deacetylase (HDAC) and demethylase functions involved in transcriptional repression [95]. Based on the differing effect of CHD4 inhibition on PAX3-FOXO1 target gene expression compared to other members of the NuRD complex, it is hypothesized that CHD4 may also act in a pathway independent of the NuRD complex. BRD4 belongs to the bromodomain and extra-terminal domain (BET) protein family in which each member contains two conserved N-terminal bromodomains. These domains are chromatin interaction modules that recognize acetylated lysines on nuclear proteins such as histones and transcription factors. BRD4 acts to recruit transcriptional regulatory complexes to the acetylated chromatin region [96]. As activation of mammalian gene expression requires a concerted action of transcription factors and chromatin modifiers [97], PAX3-FOXO1 is likely to gain access to the binding sites on its transcription targets within the chromatin landscape created by CHD4, BRD4 and their functioning complexes. Once bound to specific DNA binding motifs within target genes, PAX3-FOXO1 may also further modify the chromatin landscape to promote expression of its oncogenic targets. 

The experimental results of CHD4 and BRD4 inhibition in FP RMS cells resemble the effect of PAX3-FOXO1 depletion, providing a rationale for targeting transcriptional co-activators of PAX3-FOXO1, such as CHD4 and BRD4, as potential therapeutic targets in ARMS. However, it should be noted that the effects of BRD4 inhibitors and other epigenetic-based drugs on PAX3-FOXO1-positive RMS cell lines may also be mediated through other mechanisms. The finding of a prominent decrease in PAX3-FOXO1 mRNA expression caused by the HDAC inhibitor entinostat suggests that this drug is acting at the level of PAX3-FOXO1 gene transcription instead of PAX3-FOXO1 protein function [87]. The HDAC inhibitor JNJ-2648185 induces mitochondria-mediated apoptosis in both FP and FN cell lines [98]. JNJ-2648185 was also shown to synergistically enhance apoptotic effects of common anticancer drugs for RMS such as vincristine, actinomycin D, cyclophosphamide, etoposide and doxurubicin in both FP and FN cell lines [99,100]. Combinations of BRD4 and HDAC inhibitors also effectively and synergistically induce apoptosis in both FP and FN cell lines [101]. Thus, these inhibitors may exert antitumor effects by generally disrupting the interplay between transcription factors and the epigenome. Still, there appears to be some level of anti-growth selectivity for FP RMS cells treated with these BET and HDAC inhibitors suggesting a dominant role of transcriptional pathways involving the PAX3-FOXO1 protein and a resulting dependency of these lines on the fusion protein for cell growth and proliferation. Therefore, these inhibitors are considered as important potential drugs for treating FP RMS.

#### 3.2.3. Targeting the Acetylation of PAX3-FOXO1

Another potential therapeutic target in FP RMS is the lysine acetyltransferase (KAT) domain-containing enzyme P/CAF (p300/CBP). PAX3-FOXO1 interacts with P/CAF in FP RMS cells, and the interaction induces acetylation of PAX3-FOXO1 at lysine residues K426 and K429, which are located within the transactivation domain, resulting in increased PAX3-FOXO1 stability and transcriptional activity [86] (Figure 2). Knockdown of P/CAF or inhibition of its acetyltransferase activity by the small molecule embelin reduces PAX3-FOXO1 protein expression and suppresses growth and proliferation of FP RMS lines and xenograft tumors [86]. Embelin specifically interacts with the CoA binding pocket within the KAT domain of P/CAF, and by juxtaposing the 11-carbon alkyl chain of embelin into close contact with the C574 catalytic residue, this small molecule inhibits the KAT activity of P/CAF [102]. Of note, acetylation of the equivalent lysine on FOXO1 (K245) and a nearby residue (K262) was previously shown to enhance AKT1-mediated phosphorylation of FOXO1 and attenuate its binding affinity with cognate DNA targets [103]. In this context, it is possible that AKT is responsible for the contrasting effects of acetylation on the transcriptional activity of FOXO1 and PAX3-FOXO1; AKT1 was previously reported to phosphorylate FOXO1, leading to its cytoplasmic retention and inactivation [104], but it had no effect on the PAX3-FOXO1 fusion protein [92]. Finally, it should also be noted that P/CAF inhibition might cause additional effects in FP RMS cells. For example, P/CAF is a required cofactor for transcriptional activity of the myogenic differentiation factor MyoD, whose corresponding gene is a transcriptional target of PAX3-FOXO1. P/CAF acetylates histones 3 and 4 at the promoter sites of MyoD target genes, creating a chromatin state that activates its transcriptional activity in muscle cells [105,106]. 

### 3.3 Targeting Downstream Effectors of PAX3-FOXO1

PAX3-FOXO1 exerts its oncogenic effect through transcriptional activation of downstream targets whose expression is proposed to promote tumorigenesis. High-throughput technologies such as DNA microarray, RNA sequencing and chromatin immunoprecipitation sequencing (ChIP-Seq) have enabled generation of comprehensive signatures of downstream targets expressed in PAX3-FOXO1-expressing tumors. Several of these downstream effectors have activities that promote cancer development, such as stimulating cell proliferation and inhibiting apoptosis [16,42,71,107,108], and thus a subset of these targets may confer the tumorigenic effects of PAX3-FOXO1. These findings provide a rationale for potentially targeting one or more of these downstream targets as a potential therapeutic intervention in FP RMS. Specific inhibitors have been identified and characterized for some oncogenic targets of PAX3-FOXO1 (Table 3). These inhibitors selectively suppress cell growth and proliferation in vitro and suppress tumor growth in vivo in preclinical models of FP RMS [99,109,110,111,112,113,114,115,116]. Previously, we reviewed potential targets such as FGFR4, MYCN and MET that have been explored for FP RMS treatment [16]. Recent studies have provided more evidence regarding the effects resulting from inhibition of these targets and have identified new targets for treatment of FP RMS. We update the list of potential candidates that have been or could be explored for treatment of FP RMS patients (Table 3, [69,117,118,119,120,121,122,123]). 

In these studies of downstream targets of PAX3-FOXO1, the oncogenic activity of PAX3-FOXO1 is postulated to result from the collective effects of multiple downstream targets. Indeed, individual expression of each of these downstream targets does not recapitulate the full oncogenic effect of PAX3-FOXO1. These findings suggest that each of the selected downstream targets may be necessary but not sufficient for the oncogenic effect of PAX3-FOXO1. In contrast, as described above and in Table 3, multiple studies have reported that individual inhibition of one of several downstream targets can induce tumor regression in preclinical models. Thus, there may be limitations in the interpretation or overall validity of the experimental models utilized in these studies.

## 4. Immunotherapy Applications to Target PAX3-FOXO1 and Downstream Pathways

### 4.1. Targeting PAX3-FOXO1 by Immunotherapy

Immunotherapies such as neutralizing antibodies, cancer vaccines and T cells modified to express a chimeric antigen receptor (CAR) have been successfully applied to treat multiple types of human cancer. Efforts have been made to develop cancer vaccines that specifically target PAX3-FOXO1, a nuclear oncoprotein with potential neoantigens [146,147,148,149]. Though one study did not find a peptide antigen in the fusion breakpoint region that could effectively stimulate a cytotoxic lymphocyte response, a second study identified a peptide from the PAX3-FOXO1 breakpoint area that is able to elicit such an immune response [147,148]. In particular, this latter study pulsed autologous dendritic cells from a normal donor with the breakpoint peptide to generate a cytotoxic lymphocyte line that can lyse RMS tumor cells expressing PAX3-FOXO1 [148]. A subsequent clinical pilot study found that 39% of patients with advanced RMS or Ewing’s sarcoma receiving dendritic cells pulsed with peptides derived from the fusion breakpoint region showed measurable immune response to the corresponding fusion peptides [149]. However, the finding that all these patients were capable of developing an immune response to the influenza vaccine, which was concurrently administered, indicated that the fusion peptides were not consistently immunogenic. In addition, the immune response to the fusion peptides was found to be short-lived and was not associated with a difference in outcome. Additional technological improvements are thus needed to induce a strong, sustained and clinically effective immune response to the PAX3-FOXO1 fusion peptides in RMS patients.

### 4.2. Targeting Cell Surface Targets of PAX3-FOXO1 by Monoclonal Antibodies

In addition to targeting oncogenic effectors, some of the PAX3-FOXO1 targets, such as FGFR4, CXCR4 and IGF1R, are cell surface antigens, and thus constitute attractive molecular targets for immunotherapy. The high expression levels of these surface antigens in FP tumor cells allow a possible dose titration of immunologic agents to specifically target the cancer cells while causing minimum effects on normal body cells.

Monoclonal antibodies (MAB) are perhaps the most common and effective immunologic agents used to target cell surface antigens. These agents often act by binding to the extracellular domains of antigens, and then inhibiting ligand binding and/or promoting intracellular internalization. Several MABs against surface antigens in FP RMS have been developed and tested for their effect on RMS tumors. IGF1R MABs are very effective in inhibiting growth of RMS cell lines that express high levels of the IGF1R protein and inducing regression of xenograft tumors generated from these cell lines [150]. Several clinical trials using IGF1R MAB also recruited RMS patients [132,151] and ongoing NCT03041701; however, the low number of FP RMS patients recruited to date does not permit any conclusion about their utility for treating FP RMS. Similarly, CXCR4 MABs are efficient in suppressing cell growth, invasion and metastasis of FP RMS cells [141,152]. Neutralizing MABs targeting PDGFRα inhibit cell growth of PAX3-FOXO1-expressing cells both in vitro and in vivo [119]. Currently, several clinical trials are being conducted to evaluate the effect of MABs against these surface antigens in human cancer, and these trials include FP RMS patients (Table 3).

### 4.3. Targeting Cell Surface Targets of PAX3-FOXO1 by CAR T-Cells

Recently, T-cell-mediated immunotherapy has been developed as a novel approach for cancer treatment [153,154]. In this therapy, T cells are genetically modified to express chimeric antigen receptors (CAR) that target cell surface markers on cancer cells. Therapy with CAR T cells has been shown to be highly effective in certain types of leukemia and lymphoma; for example CAR T cells targeting CD19 have strong activity in the treatment of refractory B-cell malignancies [155,156]. In addition, other types of CAR T cell therapy are being investigated in clinical trials of hematological malignancies as well as solid tumors [156,157,158,159] and reviewed in [160,161,162]. Preclinical data suggest that T cell therapy could be employed to eradicate cells expressing surface antigens encoded by transcriptional targets of PAX3-FOXO1. In particular, T cells genetically modified to express a CAR that targets FGFR4 have been developed for treatment of RMS tumors [151]. Testing of these FGFR4-specific CAR T cells in vitro and in mouse models revealed promising effects of these T cells in killing tumor cells and suggested that these agents could be beneficial for treatment of high-risk, refractory and relapsed RMS [126,127,163]. In addition, IGFR1-specific CAR T cells were recently developed, and initial results indicated that they suppress growth of several sarcoma categories expressing this receptor in both cell line and xenograft systems [164].

## 5. Future Approaches in the Treatment of RMS

### 5.1. Inhibition of PAX3-FOXO1 Expression by Oligonucleotide-Based Technologies

Synthetic oligonucleotide-mediated targeting technologies, including antisense oligo-nucleotides (ASO), RNA interference (RNAi) and more recently CRISPR/Cas9-based genome editing, have proven to be effective strategies to inhibit in vitro expression of many cellular targets. For a difficult-to-target protein like PAX3-FOXO1, in which pharmaceutically targeted approaches may not be readily available, the oligonucleotide-based approaches appear to be desirable alternatives due to their high specificity. Although considered highly specific, these oligonucleotide-based technologies can have off-target effects, often due to inadequate sequence specificity. These effects can be detected in most cases by comparing the effects of independent reagents and performing rescue experiments.

Specific ASOs against the PAX3 gene were used successfully to inhibit PAX3-FOXO1 expression and induce apoptosis in a FP RMS cell line [165], though the number of studies using this technology for PAX3-FOXO1 suppression are very limited. The advancement in silencing approaches represented by RNAi and CRISPR technologies has contributed to the phasing out of ASO-mediated approaches. Specific RNAi approaches designed against PAX3-FOXO1 (based on isolated oligonucleotides or shRNA-containing expression constructs) have been used successfully in a number of studies in cell culture experiments and xenograft tumors. These approaches significantly decrease PAX3-FOXO1 expression, and induce myogenic differentiation, growth arrest and apoptosis in FP RMS lines [54,58].

During the last few years, CRISPR/Cas9 has become a method of choice over RNAi and ASOs in many in vitro and in vivo studies due to its relatively simple design and the ability to completely inhibit target expression compared to the uncertain degree of silencing often obtained with the other methods. The CRISPR/Cas9 construct can be delivered by viral transduction to tumor cells to abolish PAX3-FOXO1 expression in both cell culture and xenograft models of FP RMS. In particular, CRISPR-mediated knockout of PAX3-FOXO1 expression resulted in a significant regression of FP RMS xenograft tumors [166]. Similar inhibitory effects were observed using CRISPR-mediated knockout of exogenous PAX3-FOXO1 expression in a myoblast model of FP RMS [55].

Currently, specific tools targeting PAX3-FOXO1 such as RNAi or CRISPR, which can be very effective in preclinical models, are still not applicable in the clinical setting, mainly due to the lack of an efficient delivery system for human use. One proposed means for delivery involves use of nanoparticles to encase the therapeutic agent. Despite advances in manufacturing nanoparticles for use in humans, multiple structural features need to be engineered and optimized to deliver safe therapeutic doses of these nanoparticle-packed agents and to minimize clearance by the body. An encouraging finding in several recent studies suggests that endogenous nanoparticles (exosomes), nano-sized extracellular vesicles with a membrane lipid bilayer that are released by all types of body cells, can be highly effective as a delivery vehicle for clinical use. Importantly, the lipid bilayer exosomes have been found to contain immune modulating factors that protect them from being targeted by the immune system and can efficiently enter target cells [167]. A recent study provided an example in which siRNA-packaged exosomes efficiently target oncogenic KRAS in a mouse model of pancreatic cancer [168]. These studies hold promise for a new class of vehicles that can be safely and efficiently utilized to deliver specific agents for clinical use.

### 5.2. Identification of New Therapeutic Targets for RMS

A potential avenue for identifying new approaches to seemingly “undruggable” oncogenic targets is the elucidation of novel gene dependencies by synthetic lethality screening [75,169]. In the context of oncogene addiction, the gain-of-function mutations that contribute to tumorigenesis often leads to cellular dependence on the expression of another gene and thus confers tumor-specific vulnerabilities if that second gene is inhibited. For example, investigation of synthetic lethality interactions involving the RAS or MYC oncogenes has pointed to new directions for potential therapy of tumor cells whose growth is addicted to one of these intractable oncogenes [170,171,172]. Such approaches could also be considered for PAX3-FOXO1 and similar fusion oncoproteins. Recent advances in genome-scale technologies such as whole genome CRISPR-based genetic screens, which can allow comprehensive interrogations of functional genomics, are expected to escalate the identification of synthetic lethality interactions or dependencies for many oncoproteins.

## 6. Ongoing Challenges in the Development of Therapies for RMS

A challenge complicating the development of therapies targeting the fusion oncoproteins in FP RMS and other sarcomas is the relatively small number of patients afflicted with such cancers. For a tumor-specific oncoprotein in a rare disease, there is not much financial motivation for pharmaceutical companies to invest the considerable resources required for drug development to identify and optimize specific inhibitors. In contrast, oncogenic changes that are present in common tumors or in multiple tumor categories provide a larger potential market and are thus much more attractive targets for commercial drug development.

In the studies of PAX3-FOXO1 described above, more experimental data are needed to establish whether the results obtained from the selected preclinical models are sufficient to infer a similar effect in humans. It is important to acknowledge that there are clear issues involved in using long-term tumor-derived cell lines that have been selected for optimal growth in cell culture conditions. There is a body of evidence indicating genetic and epigenetic alterations in such cell lines when compared to primary human tumors. At the present time, patient-derived xenografts (PDX), which are directly established from primary human tumors and not passaged in cell culture, more faithfully reflect the behavior of the original tumors and thus appear to be a superior system for preclinical studies (reviewed in [173,174,175]). Therefore, it is recommended that the promising pre-clinical results for targeted therapeutics of FP RMS be validated in PDX models before proceeding to investigate the effect of these therapies in clinical trials. However, it must also be appreciated that these PDX studies are conducted in immunocompromised mice so that the interactions of the immune system are not investigated, and thus there is also a role for other animal models with intact immune systems to fully understand the effects of these targeted agents.

If successfully developed and implemented, therapeutic targeting of PAX3-FOXO1 may provide new opportunities for treating FP RMS patients. However, as has been found for other targeted therapies, treatment with a PAX3-FOXO1-targeting drug as a single agent will most likely fail to provide a long-term cure due to the development of tumor cells resistant to this therapy. There is certainly heterogeneity within any FP RMS tumor and there are likely to be rare cells within the population that possess an ability to evade any single therapeutic mechanism. In addition, though the FP RMS tumor cells appear to be addicted to the PAX3-FOXO1 fusion protein, we recently demonstrated in a myoblast model of FP RMS that PAX3-FOXO1-independent tumors can recur after fusion protein expression is inhibited [52]. Multidrug chemotherapy together with surgery and radiotherapy generally results in a greater long-term therapeutic impact than a single modality or agent. The combined synergistic or additive effect of multiple drugs and therapeutic modalities substantially reduces the probability of resistance and ensures that both local and distal disease are managed. Furthermore, the combination of drugs may allow reduced doses of each drug, thus avoiding toxicity associated with higher dosages of a single agent. Therefore, as with conventional cancer therapies, the combination of several drugs and treatment modalities continues to be a cornerstone in cancer therapy.

## 7. Conclusions

Molecularly targeted therapies have become a reality for treatment of many human cancers. The success is attributed to accumulated understanding of tumor genetics, including identification of cancer driver genes, specific expression signatures and essential signaling networks specific for particular groups or subgroups of cancer. Gene fusions emerge as an attractive category of cancer genes, because the chimeric molecules encoded by these gene fusions are often dominant-acting drivers of tumorigenesis and usually expressed only in tumor cells. Successful therapies have been developed to directly target several fusion proteins involved in signal transduction, such as BCR-ABL in leukemia and EML4-ALK1 in lung carcinoma. However, the development of therapeutic tools to suppress the oncogenic activity of other fusion proteins, such as fusion transcription factors, has encountered numerous challenges, including protein structural constraints and access to the nuclear localization.

Though efforts will continue to identify drugs that specifically target various tumor-specific fusion oncoproteins, investigations of the larger biological context of these fusion transcription factors has led to a larger set of therapeutic opportunities that can take advantage of pre-existing drugs. For FP RMS, an understanding of the signaling pathways controlling the biological functions of PAX3-FOXO1 has pointed to inhibitors of these pathways as promising therapeutic tools. The recent identification of kinases regulating PAX3-FOXO1 and discovery of its essential cofactors such as CHD4 and BRD4 has contributed to new conceptual strategies for targeting PAX3-FOXO1. Targeting selected downstream effectors of PAX3-FOXO1 may be another promising approach for treatment of FP RMS tumors. In addition to small molecule inhibitors of these downstream targets, a subgroup of these targets are cell surface molecules that are potential targets for immunotherapies using neutralizing antibodies or CAR T cells.

## Figures and Tables

**Table 1:** RMS subtypes and their major associated genetic alterations.

Histologic Pattern	Fusion Status	Frequency	Associated Genetic Changes	Outcome
**ARMS**	FP	~20%	● PAX3/7-FOXO1 fusions [16,41,42] ● Amplification [43,44]: 2p24 (MYCN) 12q13-14 (CDK4) 13q31-32 (MIR17HG)	Poor
	FN	~5%	● Point mutations [17]: RAS genes TP53 PIK3CA FGFR4 NF1 BCOR FBXW7 ● Loss of heterozygosity [17]: 11p15.5 9p21.3 (CDKN2A) ● Amplification [17]: 12q15 (MDM2) ● Chromosome copy number gains [45,46]: 2, 8 and 12	Good
**ERMS**		~75%		

**Table 2:** List of small molecules reported to inhibit PAX3-FOXO1 activity.

Small Molecules	Relevant Molecular Targets	References
1. Kinase inhibitors		
TWS119	GSK3	[78]
SB212763	GSK3	[78]
TDZD-8	GSK3	[78]
Fascaplysin	CDK4	[79]
PKC412	Multiple kinases including CK2 and GSK3	[19]
BI-2536	PLK1	[80]
BI-6727 (volasertib)	PLK1	[80]
Thapsigargin	Ca^2+^ATPases	[84]
2. Inhibitors of chromatin modifying complexes		
JQ1	BRD4 and BET proteins	[85]
OTX015	BRD4 and BET proteins	[85]
iBET151	BRD4 and BET proteins	[85]
iBET726	BRD4 and BET proteins	[85]
iBET762	BRD4 and BET proteins	[85]
Embelin	P/CAF	[86]
Entinostat	HDAC	[87]
SAHA	HDAC	[87]
3. Unknown inhibitors		
Fenretinide	Unknown	[88]

**Table 3:** PAX3-FOXO1 downstream targets and specific agents that could be potentially explored for treatment of FP RMS.

Genes	Targeting Agents	References	Clinical Trials ^#1^
**1. FGFR4**	Ponatinib	[124]	
	PD173074	[109,125]	
	FGF401		NCT02325739 (Phase II)
	FGFR4 MAB ^#2^	[126]	
	FGFR4 CAR T	[127]	
**2. ALK1**	Crizotinib	[116]	NCT02034981 (II)
	LDK378	[128,129]	NCT01742286 (I)
**3. IGF1R**	Ganitumab ^#2^	[130]	NCT03041701(II)
	Dalotuzumab ^#2^	[131]	NCT00694356 (I)
	R1507	[132]	NCT00642941 (II)
	Picropodophyllin	[133]	
**4. PDGFRα**	Sorafenib	[134,135]	NCT01502410 (II)
	Dasatinib	[119]	NCT00464620 (II)
	Sunitinib	[134]	NCT00474994 (II)
	Axitinib	[136]	NCT01140737 (II)
	Pazopanib	[137,138]	NCT01956669 (II)
	Olaratumab ^#2^	[139]	NCT01185964 (II)
**5. MET**	Crizotinib	[116]	NCT02034981 (II)
	Tivantinib	[140]	
**6. CXCR4**	MAB CF172 ^#2^	[141]	
**7. MYCN**	PNA-MYCN	[142]	
	Sphingosine	[143]	
**8. JARID2**	JHDM inhibitor	[144]	
**9. CB1**	AM251	[69]	
	HU210	[122]	
	THC	[122]	
**10. CPT1A**	Etomoxir	[145]	

^#1^ Clinical trials that recruit RMS patients. ^#2^ MAB, mab: monoclonal antibody.
